# Supplementary material for: Beyond Care: A Scoping Review on the Work Environment of Oncology Nurses
Source: Nurs Rep. 2025 Sep 5;15(9):324. doi: 10.3390/nursrep15090324 (PMC12472513; doi:10.3390/nursrep15090324)
Supplement: Supplementary file 1 [file nursrep-15-00324-s001.zip › nursrep-3820403-supplementary.pdf]

Table S1. Preferred Reporting Items for Systematic reviews and Meta-Analyses extension for Scoping Reviews (PRISMA-ScR) Checklist

SECTION	ITEM	PRISMA-ScR CHECKLIST ITEM	REPORTED ON PAGE #
**TITLE**			
Title	1	Identify the report as a scoping review.	1
**ABSTRACT**			
Structured summary	2	Provide a structured summary that includes (as applicable): background, objectives, eligibility criteria, sources of evidence, charting methods, results, and conclusions that relate to the review questions and objectives.	1
**INTRODUCTION**			
Rationale	3	Describe the rationale for the review in the context of what is already known. Explain why the review questions/objectives lend themselves to a scoping review approach.	1-2
Objectives	4	Provide an explicit statement of the questions and objectives being addressed with reference to their key elements (e.g., population or participants, concepts, and context) or other relevant key elements used to conceptualize the review questions and/or objectives.	2
**METHODS**			
Protocol and registration	5	Indicate whether a review protocol exists; state if and where it can be accessed (e.g., a Web address); and if available, provide registration information, including the registration number.	Not applicable
Eligibility criteria	6	Specify characteristics of the sources of evidence used as eligibility criteria (e.g., years considered, language, and publication status), and provide a rationale.	3
Information sources*	7	Describe all information sources in the search (e.g., databases with dates of coverage and contact with authors to identify additional sources), as well as the date the most recent search was executed.	3
Search	8	Present the full electronic search strategy for at least 1 database, including any limits used, such that it could be repeated.	Supplementary Material
Selection of sources of evidence†	9	State the process for selecting sources of evidence (i.e., screening and eligibility) included in the scoping review.	3
Data charting process‡	10	Describe the methods of charting data from the included sources of evidence (e.g., calibrated forms or forms that have been tested by the team before their use, and whether data charting was done independently or in duplicate) and any processes for obtaining and confirming data from investigators.	3
Data items	11	List and define all variables for which data were sought and any assumptions and simplifications made.	3
Critical appraisal of individual sources of evidence§	12	If done, provide a rationale for conducting a critical appraisal of included sources of evidence; describe the methods used and how this information was used in any data synthesis (if appropriate).	Not applicable
Synthesis of results	13	Describe the methods of handling and summarizing the data that were charted.	3
**RESULTS**			
Selection of sources of evidence	14	Give numbers of sources of evidence screened, assessed for eligibility, and included in the review, with reasons for exclusions at each stage, ideally using a flow diagram.	4

SECTION	ITEM	PRISMA-ScR CHECKLIST ITEM	REPORTED ON PAGE #
Characteristics of sources of evidence	15	For each source of evidence, present characteristics for which data were charted and provide the citations.	4
Critical appraisal within sources of evidence	16	If done, present data on critical appraisal of included sources of evidence (see item 12).	Not applicable
Results of individual sources of evidence	17	For each included source of evidence, present the relevant data that were charted that relate to the review questions and objectives.	4-11 Supplementary Material
Synthesis of results	18	Summarize and/or present the charting results as they relate to the review questions and objectives.	4-11 Supplementary Material
**DISCUSSION**			
Summary of evidence	19	Summarize the main results (including an overview of concepts, themes, and types of evidence available), link to the review questions and objectives, and consider the relevance to key groups.	12
Limitations	20	Discuss the limitations of the scoping review process.	13
Conclusions	21	Provide a general interpretation of the results with respect to the review questions and objectives, as well as potential implications and/or next steps.	14
**FUNDING**			
Funding	22	Describe sources of funding for the included sources of evidence, as well as sources of funding for the scoping review. Describe the role of the funders of the scoping review.	NA

JBI = Joanna Briggs Institute; PRISMA-ScR = Preferred Reporting Items for Systematic reviews and Meta-Analyses extension for Scoping Reviews.

* Where *sources of evidence* (see second footnote) are compiled from, such as bibliographic databases, social media platforms, and Web sites.

† A more inclusive/heterogeneous term used to account for the different types of evidence or data sources (e.g., quantitative and/or qualitative research, expert opinion, and policy documents) that may be eligible in a scoping review as opposed to only studies. This is not to be confused with *information sources* (see first footnote).

‡ The frameworks by Arksey and O'Malley (6) and Levac and colleagues (7) and the JBI guidance (4, 5) refer to the process of data extraction in a scoping review as data charting.

§ The process of systematically examining research evidence to assess its validity, results, and relevance before using it to inform a decision. This term is used for items 12 and 19 instead of "risk of bias" (which is more applicable to systematic reviews of interventions) to include and acknowledge the various sources of evidence that may be used in a scoping review (e.g., quantitative and/or qualitative research, expert opinion, and policy document).

From: Tricco AC, Lillie E, Zarin W, O'Brien KK, Colquhoun H, Levac D, et al. PRISMA Extension for Scoping Reviews (PRISMAScR): Checklist and Explanation. Ann Intern Med. 2018;169:467–473. doi: 10.7326/M18-0850.

Table S2. Search strategy

PubMed	(((((((((((((((((workplace[MeSH Terms]) OR (workplace[Title/Abstract])) OR (workplaces[Title/Abstract])) OR ("place of work"[Title/Abstract])) OR ("job site"[Title/Abstract])) OR ("job sites"[Title/Abstract])) OR ("work location"[Title/Abstract])) OR ("work locations"[Title/Abstract])) OR ("worksite"[Title/Abstract])) OR ("worksites"[Title/Abstract])) OR ("work environment"[Title/Abstract])) OR ("work environments"[Title/Abstract])) OR ("work-site"[Title/Abstract])) OR ("work-sites"[Title/Abstract])) OR ("working environment"[Title/Abstract])) OR ("working environments"[Title/Abstract])) OR ("workforce environment"[Title/Abstract])) OR ("workforce environments"[Title/Abstract])) OR ("work setting"[Title/Abstract])) OR ("work settings"[Title/Abstract])) AND ((((((nurses[MeSH Terms]) OR (nurse[Title/Abstract])) OR (Nurses[Title/Abstract])) OR ("registered nurse"[Title/Abstract]))) AND (((((((((((Neoplasms[MeSH Terms]) OR (neoplasms[Title/Abstract])) OR (neoplasm[Title/Abstract])) OR (tumor[Title/Abstract])) OR (tumors[Title/Abstract])) OR (cancer[Title/Abstract])) OR (cancers[Title/Abstract])) OR ("malignant neoplasm"[Title/Abstract])) OR ("malignant neoplasms"[Title/Abstract])) OR (malignancy[Title/Abstract])) OR (malignancies[Title/Abstract]))
Cinahl	((((((((((((((((((((((MH workplace+)) OR ((TI workplace OR AB workplace))) OR ((TI w orkplaces OR AB workplaces))) OR ((TI "place of work" OR AB "place of work"))) OR ((TI "job site" OR AB "job site"))) OR ((TI "job sites" OR AB "job sites"))) OR ((TI "work location" OR AB "work location"))) OR ((TI "work locations" OR AB "work locations"))) OR ((TI worksite OR AB worksite))) OR ((TI worksites OR AB worksites))) OR ((TI "work environment" OR AB "work environment"))) OR ((TI "work environments" OR AB "work environments"))) OR ((TI work-site OR AB work-site))) OR ((TI work-sites OR AB work-sites))) OR ((TI "working environment" OR AB "working environment"))) OR ((TI "working environments" OR AB "working environments"))) OR ((TI "workforce environment" OR AB "workforce environment"))) OR ((TI "workforce environments" OR AB "workforce environments"))) OR ((TI "work setting" OR AB "work setting"))) OR ((TI "work settings" OR AB "work settings"))) AND (((((((MH nurses+)) OR ((TI nurse OR AB nurse))) OR ((TI Nurses O R AB Nurses))) OR ((TI "registered nurse" OR AB "registered nurse"))))) AND ((((((((((((MH Neoplasms+)) OR ((TI neoplasms OR AB neoplasms))) OR ((TI neoplasm OR AB neoplasm))) OR ((TI tumor OR AB tumor))) OR ((TI tumors OR AB tumors))) OR ((TI cancer OR AB cancer))) OR ((TI cancers OR AB cancers))) O R ((TI "malignant neoplasm" OR AB "malignant neoplasm"))) OR ((TI "malignant neoplasms" OR AB "malignant

	neoplasms"))) OR ((TI malignancy OR AB malignancy))) OR ((TI malignancies OR AB malignancies)))
Scopus	((((((((((((((((((((((workplace) OR (workplace)) OR (workplaces)) OR ("place of work"))) OR ("job site")) OR ("job sites")) OR ("work location")) OR ("work locations")) OR (worksite)) OR (worksites)) OR ("work environment")) OR ("work environments")) OR (work-site)) OR (work-sites)) OR ("working environment")) OR ("working environments")) OR ("workforce environment")) OR ("workforce environments")) OR ("work setting")) OR ("work settings")) AND ((((((nurses) OR (nurse)) OR (Nurses))) OR ("registered nurse")))) AND (((((((((((Neoplasms) OR (neoplasms)) OR (neoplasm)) OR (tumor)) OR (tumors)) OR (cancer)) OR (cancers)) OR ("malignant neoplasm")) OR ("malignant neoplasms")) OR (malignancy)) OR (malignancies))

Table S3. Data extraction table

TITLE	**Al zoubi AM., Saifan AR., Alrimawi I. & Aljabery MA. "Challenges facing oncology nurses in Jordan: A qualitative study"**
YEAR OF PUBLICATION	2020
CONTEXT 1	Hospital
Context features	Oncology Hospital (Oncology and Hematology Units)
CONTEXT 2	Asia (Jordan)
OBJECTIVE(S) OF THE STUDY	To explore the challenges experienced by oncology nurses in Jordan during their daily practice and understand how these challenges affect their psychological and professional well-being
SAMPLE CHARACTERISTICS	The sample was chosen with a convenience technique, with the following selection criteria: • Possession of a diploma or bachelor's degree• At least 3 months of experience in the field of oncology• They must deal with direct care for the cancer patient. Exclusion Criteria: nurses with mental or psychological disorders or who were not involved in a care role A total of 24 nurses, 12 females and 12 males aged 23-42 (average 30.7), length of service 1-16 years (average 5.5), 15 single and 9 married, 17 with bachelor's degree and 7 with master's degree
TYPE OF STUDY	Descriptive qualitative study
ASSESSMENT TOOLS	The data were obtained through semi-structured interviews lasting between 30min-1h. The interviewers had an outline containing 9 structured questions to be proposed. They were digitally recorded and transcribed.
STATISTICAL ANALYSIS	An exploratory approach is used
CHARACTERISTICS OF THE STAIRS	/
RESULTS	The results were divided into three themes with two subcategories each. 1) The personal challenges of nurses: they develop emotional attachment to the patient and are unable to separate the personal sphere from the work sphere.2) The organizational challenges: the nursing staff cannot inform the patient about his real state of health/lack of authority; lack of personnel and material in the operating units. 3) Lack of orientation programsA stressful work environment negatively affects the quality of care and job satisfaction
OUTCOME	1) Emotional attachment to patients and not knowing how to separate private and professional life leads to an increase in stress, anxiety, guilt, sense of overwhelm, irritability, irritability and depression in nursing staff. 2) Nurses develop anxiety, depression or emotional burden in not being able to effectively communicate to the patient his current state of health; A lack of staff leads to an increase in mortality and morbidity. 3) Lack of guidance programs leads to stress and anxiety
Description of variables	1) Emotional attachment to patients is a normal part of their role, they support patients not only from the point of view of pathology but also on private matters. Some are able to control their feelings and it is essential to prevent suffering. Working with cancer patients allows them to reflect on their personal life and affects their

	home life: they do not have time for partners and children as they continue to think about the patients' conditions.2) The importance of restructuring the organizational system would lead to an improvement in one's psychological health with a consequent increase in the quality of care. There is a link between the lack of staff and the quality of care: it leads to an increased workload and a lack of competent work. Other factors that could affect the development of anxiety and depression are the paper-based documentation process and lack of material. 3) Orientation programs are essential to prepare new hires for work not only for pathologies and the use of machinery but also for psychological support. The role of experienced nurses in supporting new hires as a source of work, emotional, social and psychological support is fundamental.

TITLE	**Al-Ruzzieh MA., Ayaad O. & Hess RG. "The Role of Participation in and Effectiveness of Shared Governance Councils in the Nurses' Perception of a Professional Practice Work Environment"**
YEAR OF PUBLICATION	2022
CONTEXT 1	Hospital
Context features	Inpatient units, outpatient clinics and intensive care units of an oncology hospital
CONTEXT 2	Asia (Jordan)
OBJECTIVE(S) OF THE STUDY	Identify differences in nurses' perception of the work environment (PPWE) in relation to their participation in shared governance councils; Examine the shared perception of the effectiveness of governance councils among nurses who participate in such councils.
SAMPLE CHARACTERISTICS	The sample was chosen using the convenience technique. The only inclusion criterion was at least one year of work experience. A total of 580 nurses were selected, 241 males and 339 females, with an average age of 28.57 years and 6.21 years of experience, 279 single, 288 married and 13 divorced, 524 with a bachelor's degree and 56 with a master's degree, 88 work in intensive care, 234 in operating units and 258 in outpatient clinics. 176 people participate in at least one SG council. The participants in the SG councils are divided as follows: 92 males and 84 females, 147 with a bachelor's degree and 29 with a master's degree, 60 singles, 111 married and 5 divorced, an average age of 31.01 years, an average of 8.79 working years, 25 working in intensive care, 58 in the ward and 93 on an outpatient basis.
TYPE OF STUDY	Quantitative cross-sectional study with descriptive-correlational approach
ASSESSMENT TOOLS	Initially, an email was sent to nurses who wanted to be part of the SG council: the only eligibility criterion was the hiring of at least 1 year. A questionnaire consisting of three parts was administered: the first part concerned demographic information and membership status in the SG council (if they were part of it), the second part the administration of the PPWEI scale, the third part examines the perception of effectiveness of the SG councils through the Council Health (CH) questionnaire. The third part was administered only to nurses who had participated in at least 1 SG council.

STATISTICAL ANALYSIS	The following formula was used to calculate the sample size: Z(^2)pq/δ2, where Z represents the confidence interval (95%) and δ (0.05) the tolerance error. A descriptive statistical analysis of the demographic characteristics of the participants was carried out. The independent t-test was used to measure nurses' perceptions of PPWE among those who participate and do not participate in the SG council. Pearson's correlation coefficient was used to measure the relationship between the effectiveness of SG councils and the perception of PPWE among nurses who were members of SG hospitals.
CHARACTERISTICS OF THE STAIRS	The PPWEI (Professional Practice Work Environment Inventory) scale measures the components of the PPWE that serve to help nurse leaders in daily practice including 9 domains: cultural sensitivity, autonomy and control during practice, relationship with staff, teamwork, work motivation, conflict management, quality of patient care based on the amount of staff, material and resources, communication about patients via a Likert scale from 1 (strongly disagree) to 6 (strongly agree). The CH questionnaire is a valid and reliable quantitative assessment tool that measures the effectiveness of SG councils through 3 sub-levels: the elements of the bylaws, the work processes of the board and membership, and the preparation and support of council participants. The tool consists of 25 questions using a Likert scale from 1 (strongly disagree) to 5 (strongly agree).
RESULTS	The average score of the PPWEI is 4.76. The lowest score was obtained in the relationship with colleagues ($M = 4.19$) while the highest score in communication about the patient ($M = 4.96$). The 178 who participate in the SG councils have a higher score of the PPWEI than the non-participants ($p = 0.04$). Between the two groups, nurses participating in SG councils score higher in cultural sensitivity ($p = 0.029$), sufficient number of material staff and resources ($p = 0.023$), motivation to work ($p = 0.020$), autonomy and control in practice ($p = 0.000$), and supportive leadership ($p = 0.004$), respectively. The results show a strong and positive correlation between the mean of CH and the mean of all domains of PPWEI ($p < 0.005$), especially in cultural sensitivity ($p = 0.000$), teamwork ($p = 0.000$) and autonomy ($p = 0.000$). These results demonstrate an increase in the effectiveness of SG councils in professional freedom and in expressing concern about patient care.
OUTCOME	Being an SG accredited hospital involves an increase in nursing teamwork and collaboration, autonomy and decision-making, sense of control, job satisfaction, quality of care, work life, and resources and materials.
Description of variables	Shared governance is a shared decision-making process that increases nurses' empowerment and autonomy in patient management, increases patient satisfaction and improves the work environment. It supports freedom of expression regarding patient care, the implementation of interdisciplinary goals, advocacy for obtaining resources, and better resource management to improve the work environment.

TITLE	**Bakker D., Fitch MI., Green E., Butler L. & Olson K. "Oncology nursing: Finding the balance in a changing health care system"**
YEAR OF PUBLICATION	2006
CONTEXT 1	Hospital, territory and home care
Context features	/
CONTEXT 2	America (Canada)
OBJECTIVE(S) OF THE STUDY	Understand the experience of cancer nurses in the context of a changing health system; identify the challenges faced by oncology nurses as a result of healthcare reorganisations and changes in care models; explore the strategies nurses use to cope with change and maintain balance in their work environment
SAMPLE CHARACTERISTICS	The sample was chosen with a convenience technique, nurses were selected from clinical networks in different regions of Canada, with the following selection criteria: nurses who cared for cancer patients with at least 4 years of experience. A total of 51 nurses were selected, aged between 24-60 years (average 45.9), 73% had a three-year degree while 37% had a specialist in oncology. Most of the selected nurses work in an outpatient setting.
TYPE OF STUDY	Focused Qualitative Ethnographic Study
ASSESSMENT TOOLS	The data were obtained through unstructured telephone interviews lasting 30-60 min. Before the interviews, each participant was sent a list of possible questions that formed the interview lineup by email. They were digitally recorded and transcribed.
STATISTICAL ANALYSIS	An ethnographic approach is used. Data analysis phases were carried out: in a first phase 2 researchers analyzed 5 interviews where they obtained key topics that were used to form categories, all of which were evaluated by a third member. Two other members of the research team analyzed the remaining 51 interviews. The interviews were analyzed separately.
CHARACTERISTICS OF THE STAIRS	/
RESULTS	The results were divided into three themes with each of the subcategories: 1) The healthcare environment: identified areas of change in the work environment: the change in the patient's profile, organizational system and professional practice 2) Conflicting questions: the changes created conflict situations for professional practice, with the need to maximize the professional role and the time that is dedicated to patients was reduced3) Finding The right path: nurses' behavioral patterns, values, and beliefs to balance differences
OUTCOME	1) A change in the patient's profile leads to discontent and frustration in the patients themselves; the change in the organizational system leads to fragmentation and fragility in care; Professional change is seen as something positive as it increases nursing autonomy. The reduction of personnel. Workload, limited time, and system fragmentation increase the risk of burnout. Social support and professional autonomy can mitigate this risk. 2) The introduction of primary nursing has given greater autonomy and responsibility to nurses. 3) Having to balance expected care with the time available leads to increased stress
Description of variables	1) Changes in the patient's profile concern an increase in: the number of cancer patients to be followed, comorbidities and expectations; In the organizational

	change we notice: a decrease in staff (not only the qualified one), with an increase in the workload for tasks that do not compete. All nurses interviewed said they continue to perform these tasks to make sure the patient receives the best possible level of care. There is poor communication between hospitals, home care and other facilities. There is also a change in the level of leadership for which nurses feel abandoned, they do not have a reference figure to turn to in case of problems. The professional change is something positive because nurses have been recognized as professionals; However, other health professionals do not sufficiently recognize the importance of the role. The place where patients are managed has also changed with an increase in outpatient management. 2) Organizational changes are seen as a barrier by professionals. Nurses are not only those who provide patient care but also have research, organizational, managerial roles and organizational changes, such as staff reduction, have hindered their development. A positive change has been the introduction of primary nursing3) Nurses deal with daily conflicts by balancing system pressures with quality of care. Nurses find motivation in their ability to positively influence patients' lives, show resilience and dedication, maintaining a strong sense of satisfaction in their work due to their ability to improve patients' quality of life.

TITLE	**Bakker D., Strickland J., MacDonald C., Butler L., Fitch M., Olson K. & Cummings C. "The Context of Oncology Nursing Practice"**
YEAR OF PUBLICATION	2013
CONTEXT 1	/
Context features	/
CONTEXT 2	America (Canada)
OBJECTIVE(S) OF THE STUDY	Identify and analyze the context in which oncology nursing is practiced, the forces that influence this context and the topics that emerge from the scientific literature, describing the working environment of oncology nurses
SAMPLE CHARACTERISTICS	The search was carried out on the following databases: Academic Search Premier, Cancerlit, CINAHL Full-Text, the Cochrane Library, PsychINFO and PubMed. The inclusion criteria were: • Articles in English • Publications from 1990 to 2010• Focused on cancer nurses • The aim was to describe aspects of oncology nursing practice or the work environment.<br>29 articles were analyzed: 12 qualitative articles, 9 quantitative articles, 2 essays, 1 task force report, 1 position statement and 4 literature reviews.
TYPE OF STUDY	Supplementary audit
ASSESSMENT TOOLS	The Qualitative Findings Critical Appraisal Scale or the Checklist for Assessing the Validity of Descriptive/Correlational Studies were used to assess the quality of the articles. To evaluate the empirical studies, the objective of the study, the method, the population and the type of data collection were extracted. All the studies identified were analyzed and divided into categories.
STATISTICAL ANALYSIS	/
CHARACTERISTICS OF THE STAIRS	Qualitative studies were evaluated on: methodological congruence, research questions, data collection methodology, data analysis technique, interpretation and

	representation of results, participants' voice, evidence of the researcher's culture, theoretical and methodological influence, ethical conduct of the process and basis for the conclusions presented. Quantitative studies were evaluated on: the presence of a sample selection technique, representativeness of the sample, inclusion criteria, connection between hypothesis and theoretical framework, reliability and validity of measurements, ability to compare groups, use of appropriate statistical analyses, link between the results found and the theoretical framework and generalizability of the results. On all these 10 items, a score of 1 (yes) 0 (no or not specified) was given. Based on the evaluation score, the studies were ranked:1-3 points: low, 4-6 points: medium, 7-10 points: highAll items were rated in the medium-high quality section.
RESULTS	The context is complex and multifactorial, characterized by unique and contrasting challenges. The results were divided into five subcategories:1) The structural environment2) The world of cancer care3) The organizational climate4) The nature of nursing5) Relationship and support. The article also identifies the external and internal forces that shape the work context:• Internal forces: professional culture based on advocacy and holistic care, conflict between organizational culture (economic efficiency) and nursing culture (patient-centered)• External forces: divided in turn into social (aging and epidemiology) and political and economic (budget tightening, health reorganization)
OUTCOME	3) Increased workloads and lack of clear leadership increase job dissatisfaction, stress, burnout, lower quality of care perceived by patients and limit the possibility of professional development5) The unique relationship that is described between nurse and cancer patient is seen as a fundamental support to be able to overcome the chaos of routine
Description of variables	1) Describes the physical and structural elements such as the location, the model of care and the resources used. As a type of setting we find outpatient clinics, oncology clinics, hospices and palliative care. As a model of care, primary care or the multidisciplinary team. As it resources technologies to support patient care, such as computers. Neglect of the physical environment such as narrow spaces and temporary staff have caused a perception of carelessness towards the patient. 2) The development of new technologies and types of treatment, the aging of the population, the characteristics of patients, the increase in cancer diagnoses and the desire to move care to the home level require new knowledge and updating.3) Organizational climate means the change in the system of the OU that has caused an increase in stressful work. In general, the climate in oncology is more positive than in other departments. There is also a healthcare reorganization with budget cuts, staff reductions, and reduced leadership negatively affecting the work environment.4) Cancer nurses are subjected to pain, death, and suffering and try to balance their professional and private lives. Many nurses describe their work as motivating, complacent, and satisfying. The nursing role requires advanced skills including the management of complex care, psychological support and

	communication with patients and families.5) The oncological context is based on an intricate matrix of therapeutic, collegial and professional relationships. The professional/patient relationship is unique and is seen as a pillar. The differences between an oncological and a non-oncological context are also described: the similarities are the economic, social and political forces; The differences are the uniqueness and complexity of the oncological pathology, the settings in which care can be provided, the relationships that are established with patients and the personal growth that occurs in the work with death and suffering.

TITLE	**Berger RS., Wright RJ., Faith MA. & Stapleton S. "Compassion fatigue in pediatric hematology, oncology, and bone marrow transplant healtcare providers: An integrative review"**
YEAR OF PUBLICATION	2022
CONTEXT 1	Hospital
Context features	Oncology Hospital (Oncology, Hematology and Pediatric Bone Marrow Transplant Operating Units)
CONTEXT 2	America (America)
OBJECTIVE(S) OF THE STUDY	Identify risk factors for the development of Compassion Fatigue and evaluate possible intervention strategies to counteract it
SAMPLE CHARACTERISTICS	The research was carried out on the following databases: PubMed, Cumulative Index to Nursing and Allied Health Literature (CINAHL), PsychINFO, Web Of Sciences MEDLINE. The inclusion criteria were:• English language• Nurses in oncology, hematology and pediatric bone marrow transplantation. In total, 16 articles were selected: 3 qualitative, 6 quantitative, 3 mix-method and 4 revisions.
TYPE OF STUDY	Supplementary audit
ASSESSMENT TOOLS	This integrative review follows the methodology of Whittemore and Knalf.The John Hopkins Nursing Evidence-Based Practice (JHNEBP) model was used to assess the quality and appropriateness of the items.
STATISTICAL ANALYSIS	/
CHARACTERISTICS OF THE STAIRS	The studies were classified as either experimental or non-experimental. Experimental articles were given a classification based on 3 levels (1 to 3) while non-experimental articles were given 2 levels (4 to 5).
RESULTS	Three main results were produced:1) The high-risk population that may develop CF are:• People who have been working for less than 5 years• People who are under 40 years of age • People who work night shifts• People who have had personal histories of death or trauma2) The highest sources of stress that cause CF are: • Working with pediatric patients • Complex relationships with patients and family members, with the risk of crossing professional boundaries • Lack of staff with subsequent increase in work shifts and organizational conflicts • Conflicts with colleagues • Increased mortality and comorbidity of patients 3) Interventions to reduce compassion fatigue
OUTCOME	Compassion fatigue causes absenteeism and delays, decreases job performance and increases the rate of dismissal.

Description of variables	Paediatric professionals are at high risk of developing compassion fatigue due to the emotionally intense nature of their work.

TITLE	**Campos de Carvalho E., Muller M., Bachion de Carvalho P. & de Souza Melo A. "Stress in the Professional Practice of Oncology Nurses"**
YEAR OF PUBLICATION	2005
CONTEXT 1	University
Context features	Specific course in oncology at the University of São Paulo
CONTEXT 2	America (Brazil)
OBJECTIVE(S) OF THE STUDY	Identify the factors that can cause stress in the professional activity of oncology nurses and understand their impact on the work environment and quality of care
SAMPLE CHARACTERISTICS	The sample was chosen with a technique of convenience. A total of 35 nurses (including pediatric nurses) were selected and administered the SSPON scale. Of these, 35 nurses, 31 women and 4 men, have work experience ranging from 6 months to 10 years
TYPE OF STUDY	Descriptive quantitative study
ASSESSMENT TOOLS	The Stressor Scale for Pediatric Oncology Nurses (SSPON) has been adopted.
STATISTICAL ANALYSIS	/
CHARACTERISTICS OF THE STAIRS	The SSPON scale consists of 50 items that concern professional competence, satisfaction in the workplace, the relationship with patients and family members, therapeutic development, the relationship with colleagues and the organizational situation. The scale was translated from English to Portuguese and then retranslated to be placed inside the studio. Each item has a value between 0-10: <5 is not a stressor, between 5-6.5 is a medium stressor, between 6.5-7.5 stressors, > 7.5 is extremely stressful.
RESULTS	The results were divided according to the scale:• Moderately stressful factors (score 5.1-6.4): Working with pediatric patients with rare forms of cancer, staff play roles that do not belong to them, death, when a patient surrenders to the disease, dissatisfaction with how I personally handled the death of that patient, not having the answer ready to the doctor, parents' reactions to the diagnosis and prognosis, doing continuous examinations of terminal patients, not knowing how to help the patient, co-workers who cannot understand that I am busy and continue to give me orders, not knowing what to say to a parent after the diagnosis of the child, death of a patient who has just been diagnosed, working with an incompetent colleague• Stressors (score 6.4-7.5): I don't know how to answer a question about one of my patients, death of my favorite patient, not knowing what to say to a parent in front of the death of their child, working with a colleague who always tries to find something wrong with your work, recurrence of a patient, not having 7/7 hospital services available, not being able to give comfort to the patient, feeling that a colleague is not engaged in work, working with colleagues who do not take the initiative, seeing a family member suffer, when a colleague does not know what is happening to that patient, colleagues who send sick when I know they are

	not sick, not being comfortable with my abilities• Extremely stressful factors (score > 7.4): there is no adequate communication between nurse-patient, dealing with parents who do not trust my abilities, not being able to stay involved in situations, lack of adequate staff, watching a patient suffer and not being able to do anything, feeling that I have waited too long before having done something to be able to help the patient, not being able to complete all the work during the day, lack of material, when the manager fails to improve a work situation, making mistakes (he obtained the highest score: 8.6)
OUTCOME	Stress is mainly caused by organizational problems (lack of resources, lack of staff, poor communication), emotional aspects (suffering and death of patients) and leads to lower quality of care and professional errors.
Description of variables	The results were then divided into six categories of stressors:• Work colleagues: lack of initiative or professionalism on the part of all colleagues in relation to work, insensitivity to patients' needs and lack of collaboration• System demands: performing tasks in a competitive environment or getting jobs in an obstructive environment• Knowing what is about to happen: reflections on the secondary consequences of cancer and its treatments for patients and family members• Restriction of care: need for new techniques and skills to recognize and improve the suffering of patients and family members• Emotional demands: work experiences that disturb the emotional balance of the worker• Dying with dignity

TITLE	**Challinor JM., Alqudimat MR., Teixeira TAO. & Oldenmenger WH. "Oncology nursing workforce: challenges, solutions, and future strategies"**
YEAR OF PUBLICATION	2020
CONTEXT 1	Hospital
Context features	/
CONTEXT 2	America (America and Canada) and Europe (Holland)
OBJECTIVE(S) OF THE STUDY	Identify the challenges facing oncology nursing staff such as nurse shortages, recruitment difficulties, risky working conditions and burnout; explore practical solutions to overcome these challenges, including training programmes
SAMPLE CHARACTERISTICS	The search was carried out on the following databases: PubMed, Google Scholar, Google (for grey literature) and Cochrane. The inclusion criteria were as follows: articles published between 2010 and 2020 in English, Spanish and Portuguese
TYPE OF STUDY	Narrative Review
ASSESSMENT TOOLS	/
STATISTICAL ANALYSIS	/
CHARACTERISTICS OF THE STAIRS	/
RESULTS	In this article, we take an overview of:1) Shortage of oncology nursing staff2) Recruitment of oncology staff3) Providing specialized training4) Occupational risks5) Burnout in nurses: high level of burnout
OUTCOME	Lack of staff leads to a lower ability to personalize interventions, job dissatisfaction, stress and burnout. Burnout leads to medication errors, increased absenteeism and dismissal, increased staff turnover, and

	reduced quality of patient care.<br>A lack of leadership leads to more layoffs
Description of variables	1) The lack of oncology staff is alarming and it is becoming increasingly difficult to hire because it is a profession that is recognized as requiring on an emotional, physical and cognitive level.2) Difficulty in recruiting nurses for a low salary, lack of specialized training3) Specialized training exists only in high-income nations with gender inequality4) Occupational risks are defined as risks related to the profession such as exposure to dangerous drugs, radiation exposure, non-compliance with national safety guidelines5) Burnout is characterized by three factors: emotional exhaustion, depersonalization, and reduced personal fulfillment. It is caused by high workloads, a lack of emotional support, and the complexity of cancer care. Burnout involves fatigue, anxiety, headaches, and mental health problems. There is a relationship between the lack of nursing staff and job dissatisfaction, stress and burnout. Burnout is also related to a lack of leadership.

TITLE	**Cummings GG., Olson K., Hayduk L., Bakker D., Fitch M., Green E., Butler L. & Conlon M. "The relationship between nursing leadership and nurses' job satisfaction in Canadian oncology work environments"**
YEAR OF PUBLICATION	2008
CONTEXT 1	Hospital and territory
Context features	Inpatient operating units, outpatient clinics, palliative care
CONTEXT 2	America (Canada)
OBJECTIVE(S) OF THE STUDY	To develop and test a theoretical model of the environmental factors that influence the job satisfaction of cancer nurses and to understand how these factors contributed to the job satisfaction and retention of cancer nurses, providing indications to improve work environments and prevent burnout
SAMPLE CHARACTERISTICS	The sample was chosen with a technique of convenience. 515 nurses (500 females and 15 males) were selected with an average age of 46.43 years, 87.7% work full-time, 49.6% have a diploma while 8.3% have a master's degree and an average work experience of 22.9 years.
TYPE OF STUDY	Quantitative descriptive-correlational study
ASSESSMENT TOOLS	The data were obtained by filling out the questionnaire sent to members of the Canadian Association of Nurses in Oncology (CANO) and the Association Quebecoise des Infermieres en Oncologie (AQIO) and subsequently to nurses working in other provinces of Canada. The data were obtained using a self-report questionnaire sent by email in both English and French. The theoretical model obtained was specified as an equation model and was estimated using LISREL 8.54
STATISTICAL ANALYSIS	The Pearson chi-square test was used to determine differences in the presence of factors based on organizational characteristics and the individual nurse. Responses were divided into agree (strongly and normal) and disagree (strongly and normal). A stepwise logistic regression using the 14 items of the NWI-R describing the workforce and factors of professional practice was used to determine job satisfaction/dissatisfaction.

CHARACTERISTICS OF THE STAIRS	The questionnaire consisted of three sections:• Demographics: age, years of work experience, education and type of work environment• Characteristics of the work environment: contains 14 subsections taken from the Nursing Work Index-Revised (NWI-R). Participants were asked to affirm their level of agreement with sentences describing the characteristics of their work environment on a 4-point Likert scale. • Job characteristics: job satisfaction, perception of the care provided and the quality of care and intention to leave the workplace.
RESULTS	The results were divided into:• Characteristics of the work environment: relational leadership ensures a better perception of the staff, better support and supervision in the management of conflicts. Age and gender are not seen as factors that can influence the characteristics of the work environment.• Influence of nurses' job satisfaction: the factors of the work environment that have a significant relationship with job satisfaction are a good doctor-nurse relationship, a clear nursing model known to all workers, professional autonomy and the support of a manager, clear leadership. The key factor is the ability of nurses to positively influence patients' lives
OUTCOME	Good relational leadership and a good doctor-patient relationship create more opportunities for development, more opportunities to participate in decision-making for company policy, greater professional autonomy, better quality of care and support in innovation and conflict management. A good nurse-patient relationship causes greater patient job satisfaction.
Description of variables	A good work environment should be designed in such a way that it fosters communication and collaboration between doctors and nurses

TITLE	**Davis S., Lind BK. & Sorensen C. "A Comparison of Burnout Among Oncology Nurses Working in Adult and Pediatric Inpatient and Outpatient Settings"**
YEAR OF PUBLICATION	2013
CONTEXT 1	Hospital
Context features	Inpatient and outpatient operating units
CONTEXT 2	America (America)
OBJECTIVE(S) OF THE STUDY	Analyze the differences between the development of burnout among oncology nurses based on the types of work location, coping strategies, job satisfaction and demographic variables
SAMPLE CHARACTERISTICS	The sample was chosen with a convenience technique, with the following selection criteria: full-time workers with at least six months of work experience<br>In total, 74 nurses, 71 women and 3 men, aged 22-67 years, 71 Caucasian, 1 Hispanic, 1 Asian and 1 of an unspecified ethnicity were selected, 52 married and 22 unmarried, with a work experience of 1-21 years, 29 bachelor's graduates, 37 master's graduates, 37 work in an outpatient setting, 35 in a ward, 59 in adult oncology while 15 in pediatric oncology, 26 were in possession of oncological certifications
TYPE OF STUDY	Cross-sectional observational descriptive quantitative study
ASSESSMENT TOOLS	The theoretical framework for this study was based on Maslach's multidimensional theory of burnout. In this study, nurses were asked to fill out three

	questionnaires:• A demographic questionnaire• The Nursing Satisfaction and Retention Survey: only two questions were included• The Maslach Burnout Inventory (MBI)SPSS software version 16.0 was used to analyze the data
STATISTICAL ANALYSIS	/
CHARACTERISTICS OF THE STAIRS	Maslach's multidimensional theory of burnout states that it is characterized by three dimensions: emotional exhaustion, depersonalization, and less personal fulfillment, which are related to each other. The demographic questionnaire included information about the place of work, age, gender, ethnicity, marital status, type of shifts worked, number of years worked as a nurse, number of years worked in oncology, level of education and possession of oncology certifications. In addition, they are asked what coping mechanism they use to cope with stress: religious beliefs, spirituality, support from colleagues, support from family members, education, physical exercise. The two questions of the Nursing Satisfaction and Retention Survey that were included concern: 1) The level of satisfaction with the various factors present in their work activity with a Likert scale score from 1 (strongly agree) to 5 (strongly disagree). 2) How the factors mentioned above impact their desire to stay or change jobs with a Likert scale score from 1 (they affect me to stay) to 3 (they affect me to change). The factors in question are relationships with colleagues and the manager, quality of care provided, decision-making autonomy, work environment in general, relations with doctors, opportunities for career improvement, shiftwork, workload, salary, physical commitment and autonomy. Two additional factors were included: personal and geographical motivations that influence staying or changing jobs. The ranges of values were: 1) 16-56 points and 2) 15-45 points. Higher scores indicate lower job satisfaction and greater influence on wanting to change jobs. The Maslach Burnout Inventory (MBI) is a questionnaire consisting of 22 items divided into three subgroups: emotional exhaustion, depersonalization, and less personal fulfillment. Each item asks the compiler to rate how these statements stack up in their work life with a Likert score ranging from 0 (never) to 6 (daily). Scores for emotional exhaustion are from 0-54, 0-30 for depersonalization, and 0-48 for less personal accomplishment. The data obtained were in turn divided into categories. For emotional exhaustion 0-16 low, 17-26 moderate and >27 high. For depersonalization 0-6 low, 7-12 moderate, and >13 high. For less personal fulfillment, higher scores indicate less personal fulfillment
RESULTS	The most used coping mechanisms to combat stress are spirituality and support between colleagues. People who use spirituality are less likely to develop burnout. Another protective factor against burnout is having the right resources available to treat patients. The average values of the MBI are 19.4 for emotional exhaustion (moderate), 4.6 for depersonalization (low) and 40.1 for less personal fulfillment (high). As far as emotional exhaustion is concerned, there is a link with age ($p = 0.03$): the lower the age, the less likely it is to develop

	emotional exhaustion and with the work context: it is higher in outpatient nurses (p = 0.079). Regarding less personal fulfillment, nurses who work with adult patients have greater personal fulfillment than those who work in pediatrics (p = 0.05). There are significant correlations between burnout and job satisfaction:- Inverse correlation between emotional exhaustion and job satisfaction (p < 0.001)- High levels of emotional exhaustion increase quit rates (p < 0.001)- Factors that improve job satisfaction are positive environment, autonomy, peer support, and supervision
OUTCOME	A good relationship between colleagues is seen as a protective factor for the development of depersonalization and emotional exhaustion.<br>Burnout leads to increased stress, decreased job satisfaction, and decreased quality of performance
Description of variables	Spirituality is one of the best coping strategies to deal with stress as death is one of the main issues addressed in oncology, it is also linked to the concept of self-transcendence. In the study the level of depersonalization was low, this is very important as it means that oncology nurses have not developed cynicism and have passion for their work. However, we have less personal fulfillment because dealing with death in a pediatric patient is worse than dealing with it in adults.<br>We have higher values of emotional exhaustion in outpatient settings as nurses establish greater relationships with patients, increasing their sense of distress and "worthlessness", they are older and have more work experience.

TITLE	**Diaw M., Sibeoni J., Manolios E., Gouacide JM., Brami C., Verneuil L. & Revah-Levy A. "The Lived Experience of Work-Related Issues Among Oncology Nurses"**
YEAR OF PUBLICATION	2020
CONTEXT 1	Hospital
Context features	Operating Units, Day Surgery, Outpatient Clinic
CONTEXT 2	23 nations
OBJECTIVE(S) OF THE STUDY	To explore the work problems experienced by cancer nurses with the aim of generating new knowledge and offering solutions to improve the quality of working life and the quality of care
SAMPLE CHARACTERISTICS	The search was carried out on the following databases: MEDLINE, PsychINFO, CINAHL and SSCI. The inclusion criteria were: qualitative articles, peer-reviewed articles between 2002-2016, in English and talking about cancer nurses. The exclusion criteria were: palliative and terminal care-based studies, other health professionals, and nursing studentsIn total, 63 studies were selected.
TYPE OF STUDY	Metasynthesis
ASSESSMENT TOOLS	This review follows the 6-stage method inspired by the meta-ethnography model and adheres to the guidelines ENTREQ.La quality of the articles was assessed via the Critical Appraisal Skills Programme. The CERQual GRADE approach was used to evaluate the results found and the degree of confidence. NVivo 11 software was used to manage the data.
STATISTICAL ANALYSIS	/

CHARACTERISTICS OF THE STAIRS	The six states of the meta-ethnography model are:• Define the research question, the subjects and the types of studies to be included• Identify and select the studies• Verify the quality of the selected studies• Analyze the studies, identify the themes and compare the themes between the various studies• Create main themes and structure the synthesis• Write the summaryThe articles were analyzed by three independent authors and then two authors met to confront each other. The Critical Appraisal Skills Programme includes 10 questions, 2 screening questions about the research objective and use of qualitative methodology and 8 questions about sampling strategy, data collection, researcher reflexivity, ethical issues, data analysis, results and value of the research carried out. The CERQual GRADE assesses 4 key components: methodological limitations, relevance, consistency and adequacy of the data. According to this method, studies are rated as high, moderate, low, and very low (confidence grade). In general, the quality of the studies is high.
RESULTS	Two main themes emerged:<br>1) The relational dimension of problems related to the work environment, where the relationship between nurses and other health professionals, the relationship with the patient and his family and loneliness is investigated2) The coping strategies used to cope with problems related to the work environment, which are partnership, communication, support and training
OUTCOME	The loneliness that is experienced by nurses, the weight of family expectations are factors in the development of stress. Peer support reduces the development of stress and burnout.
Description of variables	1) There is an absence of dialogue between the nurses themselves and this is due to turnover and high workload. Many experience the fear of being judged by colleagues and being isolated. There is also a lack of communication between doctors and nurses; these are often excluded from clinical decisions with a perception of discredit. Nurses do not feel supported even by the hospital management and the manager. As far as the relationship with the patient is concerned, nurses express loneliness in the management of their problems, especially on the terminal patient. There is individual loneliness (the responsibility to manage one's own stresses and difficulties without external support) and institutional loneliness (lack of organizational support and adequate training)2) Partnership is used to cope with workplace distress. Listening and communication are used as a support in the relationship with the patient. Communication is considered fundamental at an organizational and interdisciplinary level, especially in the relationship with doctors and to allow nurses to coordinate optimally. Support, seen in a teamwork way, is useful for dealing with emotional distress.

TITLE	**Gi TS., Devi MK. & Kim EAN. "A systematic review on the relationship between the nursing shortage and nurses' job satisfaction, stress and burnout levels in oncology/haematology settings"**
YEAR OF PUBLICATION	2011

CONTEXT 1	Hospital
Context features	Operating units (oncology, haematology), outpatient clinic
CONTEXT 2	America (America e Canada) e Oceania (Australia)
OBJECTIVE(S) OF THE STUDY	To synthesize the best available evidence regarding the relationship between nursing staff shortages and the levels of job satisfaction, stress and burnout of nurses working in oncology/hematology settings
SAMPLE CHARACTERISTICS	The inclusion criteria for the studies are as follows: • Adult nurses working in an oncology setting• Studies that address the relationship between job satisfaction and lack of staff, stress and burnout• Studies that measure these elements with tools• RCT studies, non-randomized trials, cohort, descriptive and cross-sectional studies• English language• Articles from 1990 to 2010The following databases were used for the research: CINAHL, Medline, Scopus, ScienceDirect, PsychINFO (Ovid), PsycArticles (Ovid), Web of Science, The Cochrane Library, Proquest, Mednar.<br>7 descriptive and descriptive-correlational studies were selected
TYPE OF STUDY	Systematic review
ASSESSMENT TOOLS	Job satisfaction was measured with the Measure of Job Satisfaction scale, the Misener Nurse Practitioner Job Satisfaction Scale and the Likert scale.Stress was measured via the Pediatric Oncology Nurse Stressor Questionnaire.Il burnout was measured via the Maslach Burnout Inventory Scale. Critical evaluation of the studies was made using the Standardized Critical Appraisal ToolThe lack of staff was measured using the Nursing Work Index-Revised (NWI-R) Scale, the Intent to Leave Scale and the Practie Environment Scale of the Nursing Work Index (PES-NWI).
STATISTICAL ANALYSIS	/
CHARACTERISTICS OF THE STAIRS	The Misener Nurse Practitioner Job Satisfaction scale is a scale that is used to measure job satisfaction and is based on a Likert scale ranging from 0 (very dissatisfied) to 6 (very satisfied)The Measure of Job Satisfaction scale is based on a Likert scale ranging from 1 (very dissatisfied) to 5 (very satisfied). The Pediatric Oncology Nurse Stressor Questionnaire is based on a Likert scale ranging from 0 (not experienced) to 5 (extremely stressful)
RESULTS	Nurses who have a master's degree tend to have greater dissatisfaction than those with a bachelor's degree ($p = 0.023$). Nurses who work part-time have higher job satisfaction ($p = 0.024$). Nurses who work in the ward have more job satisfaction than those who work in the outpatient clinic or at home ($p = 0.016$). In general, nurses are minimally satisfied with the job (4.5, MNPJS scale) but satisfaction is negatively correlated with increasing number of hours ($p = 0.008$). 50% of nurses say they do not have enough time to do the work according to the MJS ($M = 2.5$).<br>Nurses working in Magnet hospitals experience less burnout and more job satisfaction than those working in a non-Magnet hospital ($p < 0.05$). 1/3 experience emotional exhaustion (average 3.2) and more than half feel exhausted at the end of their shift (average 3.6), 40% say they work too much during their shift (average 3.2). Emotional exhaustion is prevalent in wards that have

	understaffing. There is a two-way relationship between nursing shortage and dissatisfaction, stress, and burnout.
OUTCOME	Lack of staff involves:• Increased workload leading to failure to provide quality care<br>• Job dissatisfaction• Increased resignation• Emotional exhaustion → burnout• Stress
Description of variables	The critical factors that lead to staff shortages are overtime and overloadThose who work in the ward have a greater lack of staff than those who work in the outpatient clinic, have a greater complexity of patients and a greater bureaucratic workload

TITLE	**Gribben L. & Semple CJ. "Factors contributing to burnout and work-life balance in adult oncology nursing: An integrative review"**
YEAR OF PUBLICATION	2021
CONTEXT 1	Hospital
Context features	Operating units and outpatient clinic
CONTEXT 2	America, Europa, Asia e Oceania (Australia)
OBJECTIVE(S) OF THE STUDY	Exploring the factors that contribute to burnout and work-life balance for nurses working in adult oncology
SAMPLE CHARACTERISTICS	The inclusion criteria for the studies are as follows:• English language• Articles published between 2009-2019• Primary literature• Full text articlesThe following databases were used for the research: CINAHL, Ovid Medline, PsychINFO and Scopus.<br>20 items, 17 quantities and 3 mix methods were selected
TYPE OF STUDY	Supplementary audit
ASSESSMENT TOOLS	The RefWorks software was used as a tool.The studies were evaluated using the Mixed Methods Appraisal Tool (MMAT)
STATISTICAL ANALYSIS	/
CHARACTERISTICS OF THE STAIRS	/
RESULTS	Two main themes emerged:<br>1) "Inability to thrive": difficulty controlling burnout due to organizational causes. They are divided into three subgroups:• Increased workload and clinical setting: an increase in workload, shiftwork that is not compatible with social life, less time to provide quality care, lack of staff, cancellation of rest periods and increased patient comorbidities cause burnout. Complex clinical settings are associated with higher levels of burnout than in outpatient clinics.• Impact with colleagues: A healthy work environment is key to work well-being and satisfaction. • The culture of the work environment: organizational support, the balance between work and personal commitments and a healthy work environment are protective environments.2) Personal perspectives that influence burnout: they are divided into three subcategories:• Demographic factors: there is no correlation between social status, age and personal health. Nurses who are younger or who have anxiety and depression tend to have a higher level of burnout.• Professional characteristics: Studies are contradictory as to whether work experience and education can affect the development of burnout. More experience protects against burnout, but burnout can increase with a long career (over 15 years). Higher education can both protect against and aggravate burnout.• Personality: Strength, optimism, resilience

	and psychological flexibility are protective factors in the development of burnout, passive coping or neurosis on the other hand increase the development of burnout.
OUTCOME	Organizational culture, nurses' personal characteristics, and an increased care burden cause burnout. Positive relationships, peer support, and debriefing reduce burnout. Lack of support and clear communication increases the risk of burnout. A healthy work environment with effective leadership increases the level of quality of care provided and support, decreases job dissatisfaction and the risk of developing burnout.
Description of variables	1) The most common symptom of burnout is emotional exhaustion. Nurses who work in the office develop stress as they do not have continuity with the patient's care pathway while those who work in the ward develop burnout due to the complexity of the care provided to the patient.2) Some studies state that being married can both increase and decrease the risk of developing burnout.

TITLE	**Jarrad RA. & Hammad S. "Oncology nurses' compassion fatigue, burn out and compassion satisfaction"**
YEAR OF PUBLICATION	2020
CONTEXT 1	Hospital
Context features	Operating units (oncology emergency room, intensive care, bone marrow transplantation, medical oncology, surgical oncology, palliative care, pediatrics, solvents)
CONTEXT 2	Asia (Jordan)
OBJECTIVE(S) OF THE STUDY	Describe the levels of compassion fatigue, burnout, and compassion satisfaction among cancer nurses; determine the correlation between nurses' scores in the three subscales describing the levels of compassion fatigue, burnout, and compassion satisfaction; to investigate the correlation between nurses' scores on the three subscales and some demographic, organizational and leisure-related variables
SAMPLE CHARACTERISTICS	100 oncology nurses were selected, 49 males and 51 females with an average age of 25.7 years, unmarried, with an average work experience of 3.8 years.
TYPE OF STUDY	Quantitative descriptive-correlational study
ASSESSMENT TOOLS	Participants were asked to fill out a questionnaire lasting 10-15 min. The data were analyzed using the Arabic version of the Compassion Fatigue.
STATISTICAL ANALYSIS	Pearson's correlation coefficient and descriptive statistics were used.
CHARACTERISTICS OF THE STAIRS	The questionnaire included questions about age, work experience, gender, unit, marital status, salary, what they do in their free time with variables. These variables were the number of working days with daytime sleep, days off, vacation days, regular hobbies, and the type of transportation you use to work. The Arabic version of the Compassion Fatigue consists of 66 items rated by Likert scale from 0 to 5, compassion fatigue is rated on 23 items, compassion satisfaction on 26 items and burnout on 16 items.
RESULTS	Nurses have a low level of compassion satisfaction (M = 71.8), a moderate risk of burnout (M = 39.5) and a high level of compassion fatigue (M = 50.8). An increase in CF corresponds to a decrease in CS (p=0.003). A high level of CF is strongly associated

	with burnout (p<0.001). Hours of sleep are positively correlated with CS (p = 0.036)Dependent employee numbers are negatively correlated with CS (p = 0.021) Protective factors for the development of CF, burnout, and CS are adequate sleep hours and reduction of extra-family responsibilities.
OUTCOME	A lack of personnel, material, security and leadership, a continuous turnover of personnel, the presence of internal conflicts, lengthy accreditation processes, punitive systems and patient complexity lead to the development of compassion fatigue and burnout. Nurses with high levels of CF develop burnout.<br>A lack of sleep leads to impaired information integrity, clouded decision-making, difficulty planning care, practicing care, and monitoring effects, lower quality of care for patients, decreased motivation, increased safety risks, and psychological problems.
Description of variables	Compassion fatigue is the emotional fatigue and stress resulting from empathy with patients; Compassion satisfaction is the gratification felt in caring for patients.

TITLE	**Kamimura A., Schneider K., Lee CS., Crawford SD. & Friese CR. "Practice Environments of Nurses in Ambulatory Oncology Settings: A Thematic Analysis"**
YEAR OF PUBLICATION	2012
CONTEXT 1	Hospital
Context features	Academic cancer centers, community hospitals, and private practices
CONTEXT 2	America (America)
OBJECTIVE(S) OF THE STUDY	Examine the characteristics of the nursing work environment that contribute to providing safe, high-quality care to cancer patients, as well as promoting nurses' job satisfaction; identify environmental characteristics that hinder nurses' ability to provide effective care to patients; explore the positive and negative characteristics of the work environment that influence the job satisfaction of cancer nurses
SAMPLE CHARACTERISTICS	In total, 13 nurses were selected, all female, one of whom is non-Caucasian who work at least 16 hours in the clinic
TYPE OF STUDY	Qualitative study of thematic analysis
ASSESSMENT TOOLS	2 focus groups were conducted with a non-invasive methodology to explore participants' thoughts lasting 2 hours. All while maintaining the anonymity of both nurses and the working environment. The focus groups were recorded and filmed and transcribed.
STATISTICAL ANALYSIS	Thematic analysis of data and development of a conceptual model
CHARACTERISTICS OF THE STAIRS	To carry out the focus group, a semi-structured guide was constructed divided into four sections:• The researchers reviewed the study procedures and the issue of privacy with the participants• Each participant had the opportunity to describe their role and work environment• The moderator encouraged the participants to discuss what aspects improve patient care and to maintain job satisfaction• They explored the factors related to work environment that inhibits care and negatively affects job satisfaction
RESULTS	The results are divided into:• Work environment: workloads, support and resources - high volumes,

	patient turnover and high-intensity shifts. Unequal distribution of patient load, worsened by holidays, illness or temporary staff shortages.<br>• Managerial support: Proactive managers improve the organization of the workload.<br>• Physical resources: lack of necessary equipment, insufficient support from the pharmacy causes delays and coordination problems.• Communication with colleagues: there are ambivalent experiences for communication. • Positive and negative effects of the work environmentThe analysis produced a conceptual model that highlights two main factors for improving the quality of care:1. Supportive practice environment: manageable and equitable workloads, supportive and engaged managers, and adequate physical resources2. Effective communication: improves patient safety and satisfaction and increases nurses' job satisfactionThese factors are interconnected: poor communication and unfavorable environments lead to negative outcomes, such as delays in treatment and staff dissatisfaction.
OUTCOME	High patient volumes and uneven patient allocation result in poor quality of care and job dissatisfaction. A lack of medication or a lack of staff working in the pharmacy causes an excessive wait for the patient who has to undergo the therapy. Poor communication between caregivers and administrative staff causes errors in patient scheduling. When communication is effective, it improves patient flow and quality of care. A negative work environment involves: delays in treatment, neglect of patients' psychosocial needs, guilt in the caregiver for not paying necessary attention to the patient, injuries, medication errors, ineffective communication, and overall safety risks. A positive work environment involves: continuity of care, optimal patient flow, correct amount of material, better patient care, better organization, ideal days (seamless workflow and patients with predictable conditions facilitate optimal care) and collegiality
Description of variables	1) The workload increases when patients are not evenly distributed. Nurses say that the volunteers who help them improve this situation. Critical in this case is the role of the manager: his presence is evaluated positively while he has been evaluated negatively when they want to save costs. 2) Nurses experience communication difficulties with doctors, contrasting experiences with secretaries as they organize patients without taking into account nursing activity.3) The characteristics of the work environment that improve the quality of patient care are standardization, confluence of information, mutual respect and collegiality

TITLE	**Lagerlund M., Sharp L., Linqvist R., Runesdotter S. & Tishelman C. "Intention to leave the workplace among nurses working with cancer patients in acute care hospitals in Sweden"**
YEAR OF PUBLICATION	2015
CONTEXT 1	Hospital
Context features	Operating units
CONTEXT 2	Europe (Sweden)
OBJECTIVE(S) OF THE STUDY	Examine associations between leadership perceptions and intention to leave the workplace due to job

	dissatisfaction among cancer nurses; assess the intention to leave the workplace in relation to the proportion of cancer patients, the duration of work experience, the perception of the adequacy of the provision of cancer care and the levels of burnout; investigate the modifying effects of these factors on the association between leadership and intention to leave the workplace
SAMPLE CHARACTERISTICS	7412 oncology nurses working in medicine/surgery wards with adult patients. They have recently been divided into two groups: Specialized Cancer Care 1440 (SSC) and General Cancer Care 5972 (GCC). There are substantial differences between the two groups: SSC group nurses have been working full-time in large or university hospitals for less time than those in the GCC, they are younger than those and have targeted training.
TYPE OF STUDY	Quantitative cross-sectional study with descriptive-correlational approach
ASSESSMENT TOOLS	The data come from the Swedish component of the RN4CAST questionnaire. To assess leadership, some items from the Practice Environment Scale of the Nursing Work Index-Revised were used.The Maslach Burnout Inventory was used to assess burnout.
STATISTICAL ANALYSIS	Cronbach's alpha was used for data analysis. To estimate a statistical correlation, the Chi-square Test and the independent t-test were used. Spearman's correlation coefficient was used to investigate collinearity.
CHARACTERISTICS OF THE STAIRS	The RN4CAST consists of 118 items with questions about the perception of one's workplace, burnout, job satisfaction, professional growth, quantity and quality of care and level of staff. In this part of the questionnaire, typical questions about the Swedish environment were asked. The questionnaire includes items from the Practice Environment Scale of the Nursing Work Index-Revised regarding leadership assessment rated with a Likert scale from 1 (strongly disagree) to 4 (strongly agree).
RESULTS	1) Intention to leave their job: 1/3 of nurses said they wanted to leave their job within a year due to job dissatisfaction. 80% intend to remain in the nursing profession, but to change hospital context2) Leadership and intention to leave: Negatively perceived leadership is strongly associated with increased intention to leave3) Burnout: Nurses with high burnout show a higher intention to leave the workplace, especially in nurses in the GCC group4) Experience and training: Only 40% of SCC oncology nurses believe they have training adequate against 20% GCC. Nurses with > of 2 years of work experience are more likely to quit if they perceive negative leadership. The association between negative leadership and intention to leave is strongest among those who perceive their training as adequate.
OUTCOME	Abandonment of the workplace is due to a lack of education, burnout, absence of leadership
Description of variables	The perception of managerial support is crucial to reduce the intention to leave, especially among experienced nurses. SCC nurses are more likely to change hospitals while GCC nurses are more likely to quit the profession.

TITLE	**Liu L., Lv Z., Zhou Y., Liu M. & Liu Y. "The Mediating Effect of the Perceived Professional**

	**Benefit of New Nurses in Cancer Hospitals on the Nursing Work Environment, Psychological Resilience, and Transitional Shock: A Cross-Sectional Questionnaire Survey"**
YEAR OF PUBLICATION	2023
CONTEXT 1	Hospital
Context features	Three oncological osohedals
CONTEXT 2	Asia (China)
OBJECTIVE(S) OF THE STUDY	Investigate the relationship between the nursing work environment and transition shock in new nurses; examine the relationship between psychological resilience and transition shock in new nurses; explore the perceived mediating role of occupational benefit in the relationship between the work environment and transition shocks
SAMPLE CHARACTERISTICS	A sample of convenience was used: 200 nurses, with the following inclusion criteria:• Certificate of nursing practice• < 1 year of work experience• Voluntary participation in the study82% of the participants are women, with an average age of 21.82 years and 97% were single. 49% of participants have a bachelor's degree. 72% were only children and 40% had a salary greater than 4000 Yen.
TYPE OF STUDY	Quantitative cross-sectional study with descriptive-correlational approach
ASSESSMENT TOOLS	The STROBE checklist was followed to report the results. Questionnaires containing sociodemographic information and professional benefits, the Practice Environment Scale (PES), the Brief Resilience Scale (BRS) and the Transition Shock Scale for New Graduate Nursing (TSS-NGN) were completed
STATISTICAL ANALYSIS	A nonparametric test (Spearman's rho) was used to test the relationship between the perception of personal benefits for nurses, the nursing work environment, psychological resilience, and transitional shock.<br>SEM was used to analyze the association between the nursing work environment, psychological resilience, nurses' perceived personal benefits, and transitional shock. To assess the quality of the model, the chi-square, the Pearson index, the mean squared error, the good-of-fit index, the standardized difference and the nonnormed fit index were used. The significant level with Cronbach's alpha was selected at 0.01.<br>Mediation analysis (SPSS PROCESS macro, Model 6).
CHARACTERISTICS OF THE STAIRS	The sociodemographic questionnaire included information such as age, gender, marital status, education and other information. The professional benefits questionnaire consists of 29 items divided as follows: positive career perception (5 items), gratitude from family and friends (7 items), team membership (5 items), good relationship with the patient (6 items) and personal growth (6 items) assessed with a Likert scale from 1 (strongly disagree) to 5 (strongly agree). The Brief Resilience Scale (BRS) consists of 6 items: 3 positive and 3 negative evaluated with a Likert scale ranging from 1 (it does not describe me) to 5 (it describes me very well). The scores are divided as follows: 1.00-2.99 low elasticity, 3.00-4.30 medium elasticity and 4.31-5.00 high elasticity. The TSS-NGN includes four dimensions: shock due to organizational culture and climate, shock from knowledge and skills,

	psychological shock, and physical shock. In total, it consists of 27 items evaluated with a Likert scale from 1 (strongly disagree) to 5 (strongly agree) with a score ranging from 27-135. A high score indicates a high level of transitional shock.
RESULTS	The average score of benefits received by nurses is 46.25 with the highest score obtained in personal growth and the lowest in gratitude from friends and family. The average nursing work environment score is 95.35. The most positive dimension is the quality of nursing services The average psychological resilience score is 17.07.La the culture of the organization and climate is the dimension that scored the highest with 15.46 while the knowledge and skills dimension scored the least with 10.41The transition shock obtained an average score of 51.45 and the highest dimension was the organization and culture. The personal benefit of nurses is positively correlated with psychological resilience ($p < 0.01$) and with the nursing work environment ($p < 0.01$); is negatively correlated with transitional shock ($p < 0.01$)Nursing work environment is positively correlated with psychological resilience ($p < 0.01$) and negatively correlated with transitional shock ($p < 0.01$)Psychological resilience is negatively correlated with transitional shock ($p < 0.01$)Mediated effect of occupational benefits:- work environment → occupational benefits → transition shock: Mediated effect of 21.53%- psychological resilience → professional benefits → transition shock: mediated effect of 6.85%
OUTCOME	A good work environment increases job satisfaction, the integration of the new employee with the existing team, reduces the impact of the new role, induces the development of innovation and increases the quality of care for patients.
Description of variables	Transition shock represents the emotional and psychological distress that new nurses experience in transitioning from academic training to clinical practice. A good positive work environment provides a sense of security and belonging to new nurses, reducing the impact of transition shock. Involvement in hospital management and organizational support are crucial to foster the integration of new nurses. Resilience helps new nurses cope with stress, promoting a sense of professional benefit and mitigating transition shock. The perceived professional benefits are the sense of growth and professional satisfaction gained through work experience and represent a key factor in reducing the psychological impact of the shock and improving job satisfaction.

TITLE	**Medland J., Howard-Ruben J. & Whitaker E. "Fostering Psychosocial Wellness in Oncology Nurses: Addressing Burnout and Social Support in the Workplace"**
YEAR OF PUBLICATION	2004
CONTEXT 1	Hospital
Context features	Operating units and outpatient clinic
CONTEXT 2	America (America)
OBJECTIVE(S) OF THE STUDY	Identify psychosocial well-being and burnout prevention as key priorities for oncology nurse retention; examine

	strategies aimed at reducing stress, promoting supportive relationships in the workplace and facilitating the grieving process
SAMPLE CHARACTERISTICS	This review was done based on research articles, books, theories, journal articles, practical activity and personal experiences
TYPE OF STUDY	Descriptive study + review
ASSESSMENT TOOLS	/
STATISTICAL ANALYSIS	/
CHARACTERISTICS OF THE STAIRS	/
RESULTS	Burnout is a significant risk for oncology nurses and is characterized by emotional exhaustion, depersonalization, and a sense of ineffectiveness. The main sources of work stress are: - Suffering of patients and families- High emotional burden due to frequent bereavements- Organizational problems: heavy workloads and lack of support- Physical and mental exhaustion: difficulty separating professional and personal life- Lack of training to manage complex psychological needs in patientsThe psychosocial well-being of cancer nurses is crucial to prevent burnout and improve the quality of care.
OUTCOME	Work stress causes burnout to develop and causes nurses to leave work, absenteeism and reduces productivity.
Description of variables	The psychological impact of caring for others, if not properly recognized by leadership, involves emotional exhaustion. In general, the manager recognizes that the work environment is stressful but does not know how to recognize stressors. Specific stressors in the oncology area are caring for patients with complex cancer, complex treatments, dealing with death, intense relationship with the patient and family members, interdisciplinary conflicts, ethical issues, workload, isolation outside the work context, role conflicts, lack of control and characteristics of the work environment. Factors associated with the development of burnout are the ambiguity of one's professional role, workload, age, work difficulty, active coping strategies, and social support. The role of social networks is protective against burnout.

TITLE	**Mojarad FA., Jouybari L. & Sanagoo A. "Rocky Road Ahead Of Nursing Presence in the Oncology Care Unit: A Qualitative Study"**
YEAR OF PUBLICATION	2018
CONTEXT 1	Hospital
Context features	Operating units
CONTEXT 2	Asia (Iran)
OBJECTIVE(S) OF THE STUDY	Identify barriers to nursing presence in cancer care units; explore the organizational and environmental factors that hinder the ability of cancer nurses to provide quality care; analyze stressful working conditions and dysfunctional regulations that negatively affect nursing attendance; identify the specific challenges that nursing professionals face in the oncology setting such as workplace violence, lack of staffing, lack of attention to nurses' needs, and over-documentation
SAMPLE CHARACTERISTICS	A convenience sample was used. The only inclusion criteria were nurses who had been working for at least one year in an oncology ward. A total of 27 nurses were

	selected: 3 men and 24 women, with an age range of 26-58 years and with a work experience of 1-30 years. Of the 27 nurses, 20 are nurses, 4 managers, 1 coordinator and 2 quality service experts.
TYPE OF STUDY	Qualitative study with content analysis
ASSESSMENT TOOLS	Semi-structured interactive interviews were conducted with an average duration of 75 min. The data were analyzed using the Graneheim and Lundman method. The data have been validated using the Guba and Lincoln criteria (credibility, transferability, reliability and confirmability)
STATISTICAL ANALYSIS	/
CHARACTERISTICS OF THE STAIRS	/
RESULTS	Two main themes were found:• The difficult and stressful work environment: which in turn contains four subcategories such as violence in the workplace, lack of staff, disregard for nurses' needs and organizational injustice• Dysfunctional regulations: which in turn contains two subcategories such as excessive documentation and the need to have, at the same time, detailed documentation
OUTCOME	High levels of stress and negative emotions result in being subjected to violence in the workplace. Being subjected to violence in the workplace leads to emotional distress. The lack of staff leads to a reduction in the achievement of new skills or the improvement of the same, a lower quality of care, physical and emotional fatigue. This leads to job dissatisfaction, a stressful work environment, an increase in medication errors and a decrease in the quality of care. An increased paper workload leads to dissatisfaction at the patient level.
Description of variables	1) Most nurses say they have a bad work environment due to lack of staff and increased workload. Management sees them only as tools and ignores their motivational needs and expects them to work harder than they are paid, which leads to demotivation. The violence to which nurses are subjected is not purely physical but verbal and is carried out by the patients' families. They are subjected to violence due to a lack of particular skills or little work experience that leads to frustration and unrealistic expectations. Exposure to violence compromises nurses' morale and reduces their presence at the patient's bedside.2) Nurses believe that accreditation programs, when misimplemented, threaten quality of care as managers are more concerned with checking records than actual work. Accreditation programs have certainly increased paperwork

TITLE	**Ventovaara P., af Sandeberg M., Blomgren K. & Pergert P. "Moral distress and ethical climate in pediatric oncology care impact healthcare professionals' intentions to leave"**
YEAR OF PUBLICATION	2023
CONTEXT 1	Hospital
Context features	20 paediatric cancer centres
CONTEXT 2	Europe (Sweden, Finland, Denmark, Norway and Iceland)
OBJECTIVE(S) OF THE STUDY	To assess the perception of the ethical climate among health professionals working in the field of pediatric oncology; examine the experiences of moral distress

	lived by professionals; investigate the intention of health professionals to leave the workplace due to moral distress and ethical climate
SAMPLE CHARACTERISTICS	A convenience sampling of 384 nurses was chosen.
TYPE OF STUDY	Quantitative cross-sectional study with descriptive-correlational approach
ASSESSMENT TOOLS	Two instruments were used: the Hospital Ethical Climate Survey (HECS-S) adapted to the Swedish context and the Moral Distress Scale Revised (MDS-R). The questionnaires were completed by the participants online or on paper.<br>Focus groups were carried out with the participants.
STATISTICAL ANALYSIS	Item reliability was assessed via Cronbach's alpha with a score > 0.8 indicating good internal consistency. Likert scale results were reported via median or interquartile range (Q1-Q3). To see a correlation between the scales, Spearman's rank correlation and p-value were used (< 0.005 are considered statistically significant)
CHARACTERISTICS OF THE STAIRS	The Swedish Hospital Ethical Climate Survey-Shortened consists of 21 items evaluated by a Likert scale ranging from 1 (almost never) or 5 (almost always). 3 items concerning the relationship between nurses and nurse assistants were removed from the questionnaires of Norway and Denmark. The score goes from 18-90. Higher scores indicate positive perceptions of the ethical climate. The Swedish Moral Distress Scale - Revised consists of 26 items that describe situations that can be experienced as a source of moral distress assessed by a Likert scale ranging from 0 (never) to 4 (often). For each item, the frequency scores were modified for the intensity scores to create levels of moral distress. The score ranges from 0-416. Higher scores indicate high levels of moral distress. Two items were added to the scale to assess the willingness to dismiss.
RESULTS	1) Ethical climate: nurses report less positive perceptions than doctors with an average of 71. Relationships with colleagues are a key point in the positive ethical climate, but in some centers feelings of poor organizational continuity prevail2) Moral distress: average scores are moderate but with wide variations between individuals. Nurses report higher levels of moral distress (IQR 90) than other healthcare professionals. Nurses in pediatric oncology wards are particularly vulnerable to moral distress due to the emotional and organizational burden of the role3) Association between ethical climate and moral distress: there is a moderate negative correlation ($p < 0.001$)4) Intention to leave work: 6% of nurse workers intend to leave their jobs due to moral distress. No physician has been reported to plan to quit their jobs for this reason.
OUTCOME	Less continuity of care, lack of staff and lack of time to devote to conversations cause moral distress, moral conflicts and burnout. An ethical climate perceived as positive is associated with lower levels of moral distress. Nurses with high levels of moral distress are more likely to want to quit their jobs.
Description of variables	In general, doctors have a perception of a more favorable ethical climate and less moral distress than nurses. They experience more intense emotional and

	moral loads given their proximity to patients and the time spent in direct care. The main causes of moral distress for nurses are: understaffing, lack of continuity of care and insufficient time Nurses who tend to leave their jobs have significantly lower HECS-S scores (60) and significantly higher MDS-R scores (128)

TITLE	**Vryonides S., Papastavrou E., Charalambous A., Andreou P., Eleftheriou C. & Merkouris A. "Ethical climate and missed nursing care in cancer care units"**
YEAR OF PUBLICATION	2018
CONTEXT 1	Hospital
Context features	Operating units (oncology, hematology and hospice)
CONTEXT 2	Europe (Cyprus)
OBJECTIVE(S) OF THE STUDY	Identify the different types of ethical climate perceived by nurses working in adult cancer care units; examine the relationship between the different types of ethical climate identified and the levels of nursing care missed reported by nurses
SAMPLE CHARACTERISTICS	In total, 157 nurses were selected: 62.4% women and 37.6% men, aged 24-56, 82.8% with a bachelor's degree, 12.7% with a master's or master's degree, and 4.5% with a diploma, 66% had work experience greater than 5 years
TYPE OF STUDY	Quantitative cross-sectional study with descriptive-correlational approach
ASSESSMENT TOOLS	Participants had to fill out a demographic questionnaire, the Ethical Climate Questionnaire (ECQ-26) and the MISSCARE survey
STATISTICAL ANALYSIS	Pearson's correlation coefficient, independent t-test, and analysis of variance (ANOVA) were used to analyze the relationship between the type of perceived ethical climate and the level of missed care.
CHARACTERISTICS OF THE STAIRS	The Ethical Climate Questionnaire consists of 18 items that measure the different types of ethical climate such as caring (5 items), instrumental (4 items), independence (2 items), laws (3 items) and rules (4 items) evaluated by a Likert scale ranging from 0 (completely false) to 5 (completely true). A high score indicates a better level of ethical climate. The MISSCARE survey consists of 24 items evaluated by a Likert scale ranging from 1 (never) to 5 (always). High scores represent high levels of missed care.
RESULTS	The following types of ethical climate have been identified:- Caring: concern for the well-being of others- Laws and codes: compliance with external codes- Rules: compliance with local standards and regulations- Instrumental: decisions based on personal or organizational interest- Independence: decisions driven by personal beliefsThe climates of caring, rules and laws and codes are the prevailing ones. The ethical climate is positively related to missed care: instrumental climate and independence (more missed care)The ethical climate is negatively related to missed care: caring climate, rules and laws and codes (less missed care)According to the scale, we have an average missed care value of 2.51. Activities such as patient hygiene and emotional support were frequently omitted.

OUTCOME	The presence of an ethical climate oriented towards the common good and regulatory compliance reduces missed care.
Description of variables	Nursing managers can positively influence the ethical climate of the units by reducing missed care through the promotion of shared norms and the adoption of collaborative strategies.
